# Shorter Durations of Anti-HER2 Therapy for Patients with Early-Stage, HER2-Positive Breast Cancer: The Physician Perspective

**DOI:** 10.3390/curroncol30120763

**Published:** 2023-12-14

**Authors:** Michelle Bradbury, Marie-France Savard, Lisa Vandermeer, Lucas Clemons, Gregory Pond, John Hilton, Mark Clemons, Sharon McGee

**Affiliations:** 1 Department of Medicine, Division of Medical Oncology, The Ottawa Hospital, University of Ottawa, Ottawa, ON K1H 8L6, Canada; michelle.bradbury@medportal.ca (M.B.); msavard@toh.ca (M.-F.S.); jfhilton@toh.ca (J.H.); mclemons@toh.ca (M.C.); 2 Cancer Therapeutics Program, Ottawa Hospital Research Institute, Ottawa, ON K1Y 4E9, Canada; lvandermeer@ohri.ca (L.V.); lucas.clemons@mail.mcgill.ca (L.C.); 3 Department of Oncology, McMaster University, Hamilton, ON L8V 5C2, Canada; gpond@mcmaster.ca

**Keywords:** breast cancer, HER2, trastuzumab

## Abstract

Despite evidence from clinical trials showing the efficacy of shorter durations of therapy, most HER2-positive early breast cancer (EBC) patients receive a year of anti-HER2 therapy. A survey of Canadian oncologists was conducted online, with electronic data collection, and the analysis is reported descriptively. Measures collected included current practices with respect to the duration of adjuvant anti-HER2 therapy, perspectives on data regarding shorter durations of treatment, and interest in further trials on this subject. Responses were received from 42 providers across Canada. Half (50%, 21/42) reported having never recommended 6 months of anti-HER2 therapy. The primary reason physicians consider a shorter duration is in response to treatment-related toxicities (76%, 31/41). Most participants (79%, 33/42) expressed the need for more data to determine which patients can be safely and effectively treated with shorter durations. Patient factors such as young age, initial stage, hormone receptor status, and type of neoadjuvant chemotherapy were attributed to reluctance to offer shorter durations of treatment. Many respondents (83%, 35/42) expressed interest in participating in the proposed clinical trial of 6 months of anti-HER2 therapy. In contemporary Canadian practice, 12 months of anti-HER2 therapy remains the primary practice. Future trials are required to better define the role of shorter treatment durations.

## 1. Introduction

Adjuvant treatment with anti-human epidermal growth factor receptor 2 (HER2) therapy (such as trastuzumab and other biosimilars) for a total duration of 12 months is the current standard of care for most patients with HER2-positive early breast cancer (EBC) [1,2,3]. There is increasing interest in the appropriate use of a shorter duration of therapy [1,4,5,6,7]. This has primarily been driven by concerns regarding treatment-related toxicities (including cardiotoxicity), as well as associated costs, and the burden on hospital resources [1].

While 1 year of adjuvant trastuzumab was empirically used in most of the founding trials for anti-HER2 therapy, this was challenged when a subgroup of patients in the FinHER trial who received adjuvant trastuzumab for only 9 weeks had similar benefits to those seen in other adjuvant studies with longer durations of treatment [7,8,9]. Several trials have investigated shorter durations of anti-HER2 therapy, from 9 weeks to 6 months, with differing results [8,10,11,12,13]. However, a trend towards reduced benefit of 12 months of therapy has been noted for patients with lower risk disease [14]. A pre-planned subgroup analysis of the ShortHER study compared disease-free survival (DFS) in groups receiving sequential anthracycline–taxane combination chemotherapy plus either 12 months versus 9 weeks of trastuzumab [14]. The results suggested that a shorter duration of trastuzumab could be considered in patients with a lower disease burden, including tumor size <2 cm and fewer than three lymph nodes involved [15].

More recently, an individual patient data meta-analysis of five non-inferiority randomized controlled trials (RCT) of reduced duration, single agent, adjuvant trastuzumab in HER2-positive EBC was presented [16]. This study found that while invasive disease-free survival (iDFS) with 9 weeks of trastuzumab was not non-inferior to 12 months, 6 months of trastuzumab was non-inferior to 12 months. Indeed, continuing to 12 months following completion of 6 months of treatment provided a marginal 0.7% absolute additional benefit in iDFS [17]. Concerns remain, however, as to whether these data are applicable to all patients given that most patients enrolled in these trials had lower-risk disease, with more than 50% being lymph node negative, with tumors <2 cm in size, and who were treated with an anthracycline-based chemotherapy [11,12,13]. To date, clinical practice guidelines continue to recommend 12 months of adjuvant anti-HER2 therapy for patients with HER2-positive EBC [17,18,19].

The REthinking Clinical Trials (REaCT) program is Canada’s largest oncology pragmatic trials program and has performed several RCTs to optimize the care of patients with HER2-positive disease [20,21,22,23]. With the increased interest in a more personalized approach to the management of this disease, including the DECRESCENDO trial [NCT04675827], the REaCT team is interested in performing a novel, pragmatic clinical trial of 6 months of anti-HER2 therapy in HER2-positive EBC patients with low-risk disease, defined by the absence of tumor in the breast and lymph nodes at the time of surgery, following neoadjuvant systemic chemotherapy and anti-HER2 therapy [24]. To aid in the design of this study, we surveyed Canadian oncologists to understand current practices in the management of HER2-positive EBC, gain perspectives on shorter durations of anti-HER2 therapy, and gauge interest in future clinical trials on this subject.

## 2. Materials and Methods

Canadian oncology specialists responsible for the prescription of (neo)adjuvant anti-HER2 therapy for patients with HER2-positive EBC were surveyed. These included medical oncologists, surgical oncologists, and oncology general practitioners across Canada who were willing and able to complete the survey in English. 

The primary objectives of this survey were to identify current practices with respect to the duration of anti-HER2 therapy prescribed for patients with HER2-positive EBC and explore perspectives on data regarding shorter durations (less than 12 months) of therapy and clinical scenarios in which this would be considered. Secondly, we aimed to determine healthcare providers’ interest in a pragmatic clinical trial evaluating 6 months of anti-HER2 therapy, and the optimal design of such a study. 

Oncology physicians, nurses, and researchers with experience in survey development and the treatment of EBC were involved in the survey development [25]. The survey followed the structure provided by the Checklist for Reporting Results of Internet E-Surveys (CHERRIES) (Table S1) [26]. The electronic survey was multiple-choice, consisting of 1 question for assessment of eligibility, 4 questions to collect data on demographics, and 15 questions to explore current practices in the use of anti-HER2 therapy as well as insights and interest in research investigating shorter durations of treatment (File S1). The survey was piloted on 2 medical oncologists, 1 resident physician, and 2 non-healthcare professionals prior to circulation.

Physicians and other oncology healthcare providers across Canada were invited to participate in this study via a voluntary electronic survey. The research team used a collection of publicly available email addresses through the Canadian Association of Medical Oncologists, as well as those available to the research team. Participants received an email with a survey invitation, study information sheet, and a hyperlink to the anonymous electronic survey on Microsoft^®^ Forms (File S1) (https://www.office.com/). The Microsoft^®^ Forms software collected, stored, and aggregated the data into a Microsoft^®^ Excel (https://www.office.com/) sheet that was used for analysis. Both software programs were accessed from the hospital’s Microsoft^®^ OneDrive to ensure data were collected and stored securely. A reminder email with the electronic survey link was sent 4 weeks later to increase participation. The survey was conducted between 12 April 2023 and 29 June 2023. Completion of the survey implied consent to participate. Results of the surveys are reported according to the CHERRIES checklist [26]. The study and survey design were approved by the Ontario Cancer Research Ethics Board (OCREB, CTO 4227).

Responses were stored in a password-protected database accessible to the study team only. Data were further managed on an Excel spreadsheet and saved to a secure server at the Ottawa Hospital. Results of the survey were reported using descriptive statistics, primarily measures of frequency. We included all surveys that were submitted, even if respondents did not complete all questions. 

## 3. Results

### 3.1. Physician Characteristics

The overall response rate was 24% (42/177). The provider characteristics are shown in Table 1. Most respondents were medical oncologists (98%, 41/42), from academic institutions (83%, 35/42). Respondents were representative of a broad range of ages and years of experience, and came from across Canada, with 7/13 of the Canadian provinces represented.

### 3.2. Current Practices in the Use of Anti-HER2 Therapy

In this study, 98% (41/42) of providers reported that their use of neoadjuvant systemic therapy in HER2-positive patients was influenced by the results of the KATHERINE trial, which demonstrated that patients with residual invasive disease after completion of neoadjuvant therapy have a reduced risk of breast cancer recurrence with adjuvant trastuzumab emtansine (T-DM1) (Table 2) [27]. If funding/access were not an issue, providers said situations they would consider adjuvant dual anti-HER2 therapy, with both trastuzumab and pertuzumab, were: all HER2 patients receiving adjuvant therapy (2%, 1/42), high-risk HER2-postive patients (e.g., lymph node positive) receiving adjuvant therapy (76% 32/42) and no situations (21% 9/42). If funding/access were not an issue, situations they would consider dual anti-HER2 therapy in the neoadjuvant setting were: all HER2 patients receiving neoadjuvant therapy (33% 14/42), high-risk HER2 patients (e.g., locally advanced, inflammatory or EBC >2 cm or node positive) receiving neoadjuvant therapy (62% 26/42) and no situations (12% 5/42). If pertuzumab was prescribed in the neoadjuvant setting, 46% (17/37) said they would continue pertuzumab after surgery to a total of 12 months of treatment, whereas 54% (20/37) said they would not. Barriers to the use of pertuzumab in HER2-positive EBC identified by respondents were: lack of provincial funding (100%, 37/37), the added cost of treatment (62%, 23/37), modest or unclear clinical benefits (62%, 23/37), increased risk of toxicities (19%, 7/37), and increased healthcare resource requirements (41%, 15/37) (Table 2).

### 3.3. Insights on Shorter Durations of Anti-HER2 Therapy

The survey inquired about the frequency with which healthcare providers would recommend 6 months of anti-HER2 therapy for patients with HER2-positive EBC. Half of respondents reported that they would never recommend 6 months of anti-HER2 therapy, with 43% (18/42) stating they would recommend this less than 25% of the time (Table 3). Reasons cited by providers for prescribing 6 months of therapy in the past included: 40% (16/40) felt the data supported this approach, 25% (10/40) stated that the decision was based on patient request, 8% (3/40) prescribed a shorter duration in response to the COVID-19 pandemic, 10% (4/40) considered it due to treatment-related toxicities, 13% (5/40) cited other reasons, and 23% (9/40) stated they never recommend 6 months of anti-HER therapy (Table 3).

The survey asked which patients physicians would consider for treatment with 6 months of anti-HER2 therapy (Table 3); 76% (31/41) said patients who experience or are at risk of cardiotoxicity, 44% (18/41) said patients with specific barriers to treatment (travel time, etc.), 41% (17/41) said patients with lower-risk disease (e.g., tumor < 2 cm, minimal nodal disease), 39% (16/41) said patients who achieve a pathologic complete response (pCR) with neoadjuvant therapy, and 5% (2/41) said patients who receive maximal systemic chemotherapy. No respondents felt they would consider 6 months of anti-HER2 therapy for all patients, or for those treated with dual anti-HER2 therapy. Twelve percent (5/41) stated they would not recommend 6 months of anti-HER2 therapy for any patient.

Furthermore, participants were asked whether they think data supporting 6 months of single agent adjuvant trastuzumab can be applied to patients receiving dual anti-HER2 therapy; 21% (9/42) said yes, 33% (14/42) said no and 45% (19/42) were unsure. Participants were also asked whether they think data regarding 6 months of single agent adjuvant trastuzumab can be applied to patients who begin anti-HER2 therapy in the neoadjuvant setting; 24% (10/42) said yes, 38% (16/42) said no and 38% (16/42) were unsure (Table 3).

### 3.4. Interest in Further Research on Shorter Durations of Anti-HER2 Therapy

When asked about the need for more data/trials to determine which patients with HER2-positive EBC can be safely and effectively treated with a total of 6 months of trastuzumab (Table 4), 79% (33/42) felt that more data were needed, 17% (7/42) felt the current data were sufficient, while 5% (2/42) were unsure.

We proposed a potential clinical trial of a total of 6 months of anti-HER2 therapy for patients with HER2-positive EBC who receive neoadjuvant systemic chemotherapy and anti-HER2 therapy and achieve a pCR, with no residual invasive breast cancer found in the breast and/or axilla at the time of surgery. Of the participants, 83% (35/42) stated they would offer the study to patients, 7% (3/42) would not, and 10% (4/42) were unsure. Among those who stated they were not interested/unsure about offering the clinical trial in the preceding question (17% 7/42), the reasons cited were: 14% (1/7) believed there are already enough data to support 6 months of anti-HER2 therapy, 14% (1/7) felt that 6 months of treatment are not sufficient, 43% (3/7) considered other clinical trials focusing on personalized therapy for HER2-positive EBC more important, and 43% (3/7) cited other reasons.

The reasons why participants were reluctant to offer the trial to patients who achieved a complete response included: initial clinical stage (55% 23/42), young age (33% 14/42), type of chemotherapy received neoadjuvantly (29% 12/42), hormone receptor status (17% 7/42), non-use of pertuzumab in the neoadjuvant setting (7% 3/42), and other (5% 2/42). Twenty-nine percent (12/42) had no concerns offering the proposed trial to patients (Table 4).

Finally, providers were asked what potential lowering in 3-year DFS they would be willing to accept with a total of 6 months of HER2 therapy; 26% (11/42) said they would not accept any difference, 21% (9/42) said a 1% difference, 36% (15/42) said a 2% difference, 14% (6/42) said 3% difference, and 2% (1/42) said a 4% difference (Table 4).

## 4. Discussion

The choice of providing 1 year of adjuvant trastuzumab in the original HER2-positive EBC studies was not evidence-based [1,3,5]. While an individual patient data meta-analysis of five non-inferiority RCTs showed that iDFS with 6 months of adjuvant trastuzumab was non-inferior to 12 months, a 12-month duration remains a common “standard of care” [11,12,13,16,17,18,19]. Despite the cost, toxicity, inconvenience, and likely minimal therapeutic benefit of 12 months of treatment, our survey showed that 50% of respondents never recommend 6 months of anti-HER therapy and that 12 months of treatment remains the primary practice. Considering the increased interest in so-called *Common Sense Oncology* and the move towards treatment approaches that prioritize patients’ needs with treatments that improve survival and quality of life, this survey is important as it helps identify the reasons for current practices, in addition to potential clinical trials that could lead to more rational approaches to treatment [28,29].

Firstly, most of the data supporting anti-HER2 therapy in EBC come from the adjuvant setting, particularly data evaluating shorter durations of anti-HER2 therapy, with neoadjuvant therapy traditionally reserved for patients with locally advanced or inflammatory disease. However, the seminal KATHERINE study established NAT as the standard of care for patients with HER2-positive EBC and ≥T1c, N0 clinical disease, by demonstrating a significant improvement in iDFS for patients with residual disease who completed treatment with 14 cycles of T-DM1 following surgery [27]. Indeed, 98% (41/42) of survey respondents agreed that their use of neoadjuvant therapy (NAT) had changed following the results of this trial. Similarly, most respondents (76%, 32/42) were either unsure, or did not agree, that current data on shorter durations of adjuvant anti-HER2 therapy can be applied to patients who are treated in the neoadjuvant setting.

Uncertainty also remains as to which patients are appropriate for treatment with shorter durations of anti-HER2 therapy, and indeed none of the respondents felt that 6 months of anti-HER2 therapy should be considered for all HER2-positive EBC patients. Data largely support shorter durations of anti-HER2 therapy in patients with a lower disease burden (e.g., tumors ≤ 2 cm, with minimal nodal disease), which made up the majority of patients included in trials evaluating shorter durations of adjuvant trastuzumab [14]. However, neoadjuvant therapy allows patients to be risk stratified based on their response to upfront systemic therapy. Indeed, HER2-positive EBC patients who achieve a pCR with NAT have an excellent prognosis, with recent data from the I-SPY Clinical Trials Consortium showing 5- and 10-year event-free survival (EFS) rates of 93–94% and 90–91%, respectively [30]. We therefore believe that these patients with low-risk disease, as defined by their response and sensitivity to upfront systemic treatment, are an excellent population in which to consider a more personalized approach for shorter durations of anti-HER2 therapy [31].

Although there are many barriers to the use of pertuzumab in HER2-positive EBC, primarily related to a lack of funding in Canada, it is also unclear how to incorporate it in the neoadjuvant setting. Most respondents felt that dual anti-HER2 therapy should only be considered for high-risk patients (e.g., locally advanced, inflammatory or EBC >2 cm or node positive) in the neoadjuvant setting (62%, 26/42). Practices regarding the duration of neoadjuvant pertuzumab differed, with only 46% (17/37) stating they continued treatment for a total of 12 months. Most respondents were either unsure, or did not agree, that data supporting 6 months of single agent adjuvant trastuzumab could be applied to patients receiving dual anti-HER2 therapy (78%, 23/42). Interestingly, none of the respondents identified treatment with dual anti-HER2 therapy as a factor for considering a patient for 6 months of anti-HER2 targeted therapy.

The need for more research to address the current gaps in clinical knowledge, which are limiting the adoption of shorter durations of anti-HER2 therapy in EBC, was supported by the majority of respondents (33/42, 79%). Future trials will need to incorporate current practices, including neoadjuvant treatment, anthracycline-free chemotherapy backbones, and dual anti-HER2 therapy, with a greater emphasis on personalized treatment driven by clinical biomarkers and tumor profiling, in addition to traditional clinicopathologic risk stratification. However, performing these trials will be challenging in the face of rapid treatment advances and competing clinical trials in a small patient population. Furthermore, de-escalation studies are facing the added barrier of physician and patient perceptions of treatment withdrawal. There is non-negligeable toxicity associated with overtreatment that cannot be denied and de-escalation studies should be instead referred to as optimization studies, to eliminate the negative connotation associated with the “de-escalation” terminology. Indeed, enrollment to one such trial, DECRESCENDO (NCT04675827), which is investigating a simplified neoadjuvant chemotherapy backbone with fewer side effects for patients with HER2-positive EBC, was recently suspended due to serious concerns about the impact of slow recruitment on the robustness of the scientific rationale and study financial provisions [24].

However, this study does have several important limitations. These include the small sample size composed of physicians from a single country, with a publicly funded healthcare system. All surveys were included, despite some being incomplete from the participants, which could introduce non-response bias. However, the data obtained do highlight ongoing uncertainties regarding the optimal duration of anti-HER2 therapy in EBC that will help investigators design appropriate trials to address these issues. In addition, this is the only survey we are aware of that has addressed barriers and concerns regarding shorter durations of anti-HER2 therapy in EBC. A future publication will present the patient perspective on this important clinical question and will include important insights into patients’ assessment of the risk–benefit ratio of treatment.

## 5. Conclusions

It is evident that despite the minimal disease-free and overall survival benefits of 12 versus 6 months of anti-HER2 therapy for most patients, along with the definite increase in treatment-related toxicities and the financial impact, few physicians are using shorter durations of anti-HER2 therapies. While conducting clinical trials in this patient population is challenging, we owe it to our patients to perform studies that can optimize their care in a more personalized and patient-centered manner.

## Figures and Tables

**Table 1:** Baseline provider characteristics.

Characteristic	N	N (%)
**Provider Type**	42	
Medical oncologist		41 (98)
Internist with interest in oncology		1 (2)
**Years in Practice**	42	
<5 years		9 (21)
5–10 years		13 (31)
10–20 years		12 (29)
>20 years		8 (19)
**Work Setting**	42	
Academic (teaching) hospital		35 (83)
Non-academic (community) hospital		7 (17)
**Province**	42	
Western Canada (British Columbia, Alberta, Saskatchewan, Manitoba)		11 (26)
Ontario		26 (62)
Eastern Canada (Quebec, Nova Scotia)		5 (12)

**Table 2:** Current practices in the use of anti-HER2 therapy.

Survey Question	N	N (%)
**Has your use of neoadjuvant systemic therapy in HER2 positive patients changed because of the results of the Katherine trial [27]?**	42	
Yes		41 (98)
No		0 (0)
Unsure		1 (2)
**If funding/access were not an issue, in what situations would you consider dual anti-HER2 therapy in the adjuvant setting (select all that apply)?**	42	
All HER2 patients receiving adjuvant therapy		1 (2)
High-risk HER2 patients receiving adjuvant therapy		32 (76)
None		9 (21)
**If funding/access were not an issue, in what situations would you consider dual anti-HER2 therapy in the neoadjuvant setting (select all that apply)?**	42	
All HER2 patients receiving adjuvant therapy		14 (33)
High-risk HER2 patients receiving adjuvant therapy		26 (62)
None		5 (12)
**If you prescribe pertuzumab in the neoadjuvant setting, do you typically continue treatment after surgery to a total of 12 months of treatment?**	37	
Yes		17 (46)
No		20 (54)
**What are the barriers to the use of pertuzumab in early stage HER2 positive breast cancer (select all that apply)?**	37	
Lack of funding		37 (100)
Added cost of treatment		23 (62)
Modest or unclear benefit		23 (62)
Increased risk of toxicities		7 (19)
Increased healthcare resource requirements		15 (41)

**Table 3:** Insights on shorter durations of anti-HER2 therapy.

Survey Question	N	N (%)
**How frequently would you recommend 6-months of anti-HER2 therapy for patients with early stage HER2 positive breast cancer?**	42	
>75% of the time		2 (5)
50–75% of the time		0 (0)
25–50% of the time		1 (2)
<25% of the time		18 (43)
Never recommend 6-months of HER2 therapy		21 (50)
**For what reasons have you prescribed 6-months of anti-HER2 therapy (select all that apply)?**	40	
I feel the data supports the adoption of 6-months of adjuvant trastuzumab for some patients		16 (40)
I have prescribed 6-months of therapy at patients request/preference		10 (25)
I have prescribed 6-months of therapy during the COVID-19 pandemic to help reduce healthcare exposure for patients, or reduce demands on resources		3 (8)
Toxicity		4 (10)
I never recommend 6-months of anti-HER2 therapy		9 (23)
Other:		5 (13)
Elderly patients		1 (20)
Cardiotoxicity		2 (40)
Duration not shortened due to lack of evidence		2 (40)
**Which patients would you consider for treatment with 6-months of anti-HER2 therapy (select all that apply)?**	41	
All early stage HER2 positive patients should be considered for 6-months of HER2 therapy		0 (0)
Patients with lower risk disease should be considered e.g., small tumours (≤2 cm), minimal nodal disease (0–3 nodes positive)		17 (41)
Patients who experience (or are at increased risk of) toxicities e.g., cardiotoxicity		31 (76)
Patients who received maximal systemic chemotherapy including an anthracycline or an anthracycline-free regimen such as docetaxel and carboplatin		2 (5)
Patients who were treated with trastuzumab and pertuzumab		0 (0)
Patients who achieve a pathologic complete response (pCR) with neoadjuvant therapy		16 (39)
Patients with specific barriers to treatment e.g., increased travel time to the cancer centre		18 (44)
I would not recommend 6-months of anti-HER2 therapy for any patient		5 (12)
**Do you think data supporting 6-months of single agent adjuvant trastuzumab can be applied to patients receiving dual anti-HER2 therapy?**	42	
Yes		9 (21)
No		14 (33)
Unsure		19 (45)
**Do you think data regarding 6-months of single agent adjuvant trastuzumab can be applied to patients who begin anti-HER2 therapy in the neoadjuvant setting?**	42	
Yes		10 (24)
No		16 (38)
Unsure		16 (38)

**Table 4:** Evaluating interest in further research on shorter durations of anti-HER2 therapy.

Survey Question	N	N (%)
**Do you feel more data/trials are needed to determine which patients with early stage HER2 positive breast cancer can be safely and effectively treated with a total of 6-months of trastuzumab?**	42	
Yes		33 (79)
No		7 (17)
Unsure		2 (5)
**We propose a clinical trial of a total of 6-months of anti-HER2 therapy in patients who receive neoadjuvant systemic chemotherapy and anti-HER2 therapy and achieve a pCR at the time of surgery. Would you offer this study to your patients?**	42	
Yes		35 (83)
No		3 (7)
Unsure		4 (10)
**For what reasons are you not interested in a clinical trial of 6-months of anti-HER2 therapy in patients who receive neoadjuvant systemic chemotherapy and anti-HER2 therapy and achieve a pCR at the time of surgery (select all that apply)?**	7	
I think there is already enough data to support 6-months of HER2 targeted therapy		1 (14)
I do not think 6-months of HER2 targeted therapy is sufficient treatment in this setting		1 (14)
I think there are more important clinical trials to be conducted around personalized therapy in early stage HER2 positive e.g., de-escalation of (neo)adjuvant chemotherapy		3 (43)
Other:		3 (43)
Depends on baseline clinical risk		1 (33)
Insufficient evidence		1 (33)
Depends on the trial design and goals		1 (33)
**Are there any reasons that may make you more reluctant to offer this trial to a patient that achieved a pCR (select all that apply)?**	42	
No		12 (29)
Young age		14 (33)
Initial clinical stage		23 (55)
Hormone receptor status		7 (17)
Type of chemotherapy received neoadjuvantly		12 (29)
Non-use of pertuzumab in the neoadjuvant setting		3 (7)
No response		2 (5)
**Data show that HER2 positive patients who achieve a pCR at the time of surgery after neoadjuvant systemic chemotherapy and anti-HER2 therapy have an excellent prognosis (3 yr Disease Free Survival (DFS) 96%). If these patients were to be considered for a total of 6 instead of 12 months of anti-HER2 therapy (including that received in neoadjuvant period), what potential lowering in 3 yr DFS would you be willing to accept with a total of 6-months of anti-HER2 therapy?**	42	
I would not be willing to accept any difference (i.e., 3 yr DFS 96%)		11 (26)
1% (i.e., 3 yr DFS 95%)		9 (21)
2% (i.e., 3 yr DFS 94%)		15 (36)
3% (i.e., 3 yr DFS 93%)		6 (14)
4% (i.e., 3 yr DFS 92%)		1 (2)

## Data Availability

The data presented in this study are available on request from the corresponding author.

## Supplementary Materials

The following supporting information can be downloaded at: https://www.mdpi.com/article/10.3390/curroncol30120763/s1, Table S1: Checklist for Reporting Results of Internet E-Surveys (CHERRIES); File S1. Survey.
