# Supplementary material for: Shorter Durations of Anti-HER2 Therapy for Patients with Early-Stage, HER2-Positive Breast Cancer: The Physician Perspective
Source: Curr Oncol. 2023 Dec 14;30(12):10477–87. doi: 10.3390/curroncol30120763 (PMC10742686; doi:10.3390/curroncol30120763)
Supplement: Supplementary file 1 [file curroncol-30-00763-s001.zip › File S1. Survey.pdf]

Evaluating physician perspectives on shorter durations of HER2-targeted therapy in patients with early-stage, HER2 positive breast cancer (REaCT-HER TIME Survey)

Survey for Health Care Providers

The purpose of the questionnaire is to learn about current physician practices with respect to the duration of HER2 targeted therapy prescribed for patients with HER2 positive early breast cancer (EBC), perspectives on data regarding shorter durations (<12 months) of HER2 therapy, and interest in further clinical trials on this subject.

Thank you for participating in this questionnaire. All responses are anonymous, and it is estimated to take approximately 5-10 minutes to complete all questions. Completion of the survey implies consent to participate.

Assessment of Eligibility:

1. Do you prescribe systemic cancer therapy for patients with early-stage breast cancer?
   a) Yes
   b) No

IF YOU ANSWERED NO TO THIS QUESTION, PLEASE STOP THE SURVEY HERE.

Demographic Information:

2. What is your clinical profession?
   a) Medical oncologist
   b) Surgical oncologist
   c) Nurse practitioner
   d) GP oncologist
   e) Other, please specify: ________________________________

3. How many years have you been in clinical practice?
   a) <5yrs
   b) 5-10yrs
   c) 10-20yrs
   d) >20yrs

4. In what setting do you work?
   a) An academic (teaching) hospital
   b) A non-academic (community) hospital
   c) Private practice
   d) Other; please specify: ___________________________________

5. In which province or territory do you work?
   a) Alberta
   b) British Columbia
   c) Manitoba
   d) New Brunswick
   e) Newfoundland and Labrador
   f) Northwest Territories
   g) Nova Scotia
   h) Nunavut
   i) Ontario
   j) Prince Edward Island
   k) Quebec
   l) Saskatchewan
   m) Yukon

Current Practices in the use of HER2 Therapy:

6. The Katherine clinical trial evaluated trastuzumab emtansine (T-DM1) versus standard trastuzumab based therapy as adjuvant therapy in patients with early stage HER2-positive breast cancer who had residual tumour present in the breast and/or axilla following preoperative therapy. The risk of recurrent invasive breast cancer or death was 50% lower with adjuvant T-DM1 than with trastuzumab. Following these results, guidelines recommend consideration of neoadjuvant systemic therapy (NAT) for patients with HER2 positive breast cancer stage T1cN0 (tumour size 10-20mm) and above.

   Has your use of NAT in HER2 positive EBC patients increased because of this data?
   a) Yes
   b) No
   c) I don't know

7. If funding/access were not an issue, in what situations would you consider dual HER2 therapy in the ADJUVANT setting (select all that apply)?
   a) All HER2 patients receiving adjuvant treatment
   b) High-risk patients receiving adjuvant treatment e.g. lymph node positive
   c) I do not OR would not prescribe dual HER2 therapy for early stage HER2 positive breast cancer.

8. If you chose C, please explain why? ______________________________

____

9. If funding/access were not an issue in what situations would you consider dual HER2 therapy in the NEOADJUVANT setting (select all that apply)?
   a) All HER2 patients receiving neoadjuvant treatment
   b) High-risk neoadjuvant patients e.g. locally advanced, inflammatory or EBC >2cm or node positive
   c) I do not OR would not prescribe dual HER2 therapy for early stage HER2 positive breast cancer.

10. If you chose C, please explain why? ______________________________

____

11. If you prescribe pertuzumab in the neoadjuvant setting, do you typically continue treatment after surgery to a total of 12 months of treatment?
    a) Yes
    b) No

12. If no, how long do you continue pertuzumab and why? ___________________

____

13. What do you see as barriers to the use of pertuzumab in early stage HER2 positive breast cancer (select all that apply)?
    a) Lack of funding for treatment
    b) Added cost of treatment
    c) Modest or unclear benefit
    d) Increased risk of toxicities e.g. cardiotoxicity

e) Increased healthcare resource requirements e.g. treatment time
f) I don't see any barriers to the use of pertuzumab in HER2 positive EBC
g) Other, please specify: ____

Insights on Shorter Durations of HER2 Targeted Therapy:

Data from a patient level meta-analysis of several non-inferiority trials suggests that there is no significant difference in breast cancer outcomes with 6 rather than 12 months of single agent adjuvant trastuzumab, but side effects are reduced, specifically cardiotoxicity.

Earl HM, Hiller L, Dunn JA, Conte PF, D'Amico R, Guarneri V et la. Individual patient data meta-analysis of 5 non-inferiority RCTs of reduced duration single agent adjuvant trastuzumab in the treatment of HER2 positive early breast cancer. ESMO Congress 2021. Annals of Oncology (2021) 32 (suppl_5): S1283-S1346. 10.1016/annonc/annonc741

14. How frequently would you recommend 6 months of HER2 therapy for patients with early stage HER2 positive breast cancer?
    a) >75% of time
    b) 50-75% of time
    c) 25-50% of time
    d) <25% of time
    e) I never recommend 6 months of HER2 therapy

15. For what reasons have you prescribed 6 months of HER2 targeted therapy (select all that apply)?
    a) I feel the data supports the adoption of 6 months of adjuvant trastuzumab for <u>all patients</u>.
    b) I feel the data supports the adoption of 6 months of adjuvant trastuzumab for <u>some patients</u>.
    c) I have prescribed 6 months of therapy during the COVID-19 pandemic to help reduce healthcare exposure for patients, or reduce demands on resources e.g. staffing, PPE, etc.
    d) I have prescribed 6 months of therapy at patients request/preference.
    e) I would not recommend 6 months of HER2 therapy for any patient
    f) Other, please specify: ____

16. Which patients would you consider for treatment with 6 months of HER2 targeted therapy (select all that apply)?
    a) All early stage HER2 positive patients should be considered for 6 months of HER2 therapy
    b) Patients with lower risk disease should be considered e.g. small tumours (≤ 2cm), minimal nodal disease (0-3 nodes positive)
    c) Patients who experience (or are at increased risk of) toxicities e.g. cardiotoxicity
    d) Patients who received maximal systemic chemotherapy including an anthracycline or an anthracycline-free regimen such as docetaxel and carboplatin
    e) Patients who were treated with trastuzumab and pertuzumab
    f) Patients who achieve a pathologic complete response (pCR) with neoadjuvant therapy
    g) Patients with specific barriers to treatment e.g. increased travel time to the cancer centre
    h) I would not recommend 6 months of HER2 therapy for any patient
    i) Other, please specify: ____

17. Do you think data supporting 6 months of single agent adjuvant trastuzumab can be applied to patients receiving dual HER2 targeted therapy?
    a) Yes
    b) No
    c) Unsure

18. Do you think data regarding 6 months of single agent adjuvant trastuzumab can be applied to patients who begin HER2 therapy in the neoadjuvant setting?
    a) Yes
    b) No
    c) Unsure

Interest in Further Research on Shorter Durations of HER2 Targeted Therapy:

19. Do you feel more data/trials are needed to determine which patients with early stage HER2 positive breast cancer can be safely and effectively treated with a total of 6 months of trastuzumab?

a) Yes
b) No
c) Unsure

20. Data show that HER2 positive patients that achieve a pathologic complete response (pCR) to upfront systemic therapy have an excellent prognosis. We propose a clinical trial of a total of 6 months of HER2 therapy in patients who receive neoadjuvant systemic chemotherapy and HER2 therapy and achieve a pCR at the time of surgery. Would you offer this study to your patients?
    a) Yes
    b) No
    c) Unsure

21. If no, or unsure, can you tell us why (select all that apply)?
    a) I think there is already enough data to support 6 months of HER2 targeted therapy.
    b) I do not think 6 months of HER2 targeted therapy is the sufficient treatment in this setting.
    c) I think there are more important clinical trials to be conducted around personalized therapy in early stage HER2 positive e.g. de-escalation of (neo)adjuvant chemotherapy
    d) Other, please specify: ____

22. Are there any reasons that may make you more reluctant to offer this trial to a patient that achieved a pCR (select all that apply)?
    a) No
    b) Young age
    c) Initial clinical stage, such as larger tumor size or multiple lymph nodes enlarged pre-treatment
    d) Hormone receptor status e.g. HR positive
    e) Type of chemotherapy received neoadjuvantly e.g. a de-escalated regimen likely weekly taxol
    f) Non-use of pertuzumab in the neoadjuvant setting
    g) Other, please specify: ____

23. Data show that HER2 positive patients who achieve a pCR at the time of surgery after upfront neoadjuvant systemic chemotherapy and HER2 therapy have an excellent prognosis (3yr Disease Free Survival (DFS) 96%). If these patients were to be considered for a total of 6 instead of 12 months of HER2 therapy (including that received in neoadjuvant period), what potential lowering in 3yr DFS would you be willing to accept with a total of 6 months of HER2 therapy?
    a) I would not be willing to accept any difference (i.e. 3yr DFS 96%)
    b) 1% (i.e. 3yr DFS 95%)
    c) 2% (i.e. 3yr DFS 94%)
    d) 3% (i.e. 3yr DFS 93%)
    e) 4% (i.e. 3yr DFS 92%)

Thank you for completing this survey.
